# Tsunami deposits in Tunisia contemporaneous of the large 365 CE Crete earthquake and Mediterranean Sea catastrophic event

**DOI:** 10.1038/s41598-024-53225-7

**Published:** 2024-02-24

**Authors:** Nejib Bahrouni, Mustapha Meghraoui, Hafize Başak Bayraktar, Stefano Lorito, Mohamed Fawzi Zagrarni, Alina Polonia, Nabil Bel Mabrouk, Fekri Kamoun

**Affiliations:** 1 Office National des Mines, Tunis, Tunisia; 2 ITES, CNRS-UMR 7063, Université de Strasbourg, 67084 Strasbourg, France; 3 Istituto Nazionale di Geofisica e Vulcanologia (INGV), Rome, Italy; 4 Institut Supérieur des Sciences et Techniques Des Eaux, de Gabès, Gabès, Tunisia; 5 ISMAR-CNR (Istituto di Scienze Marine), Via Gobetti 101, 40129 Bologna, Italy; 6 Institut National du Patrimoine, Tunis, Tunisia; 7 Faculté des sciences de Sfax, universite de Sfax, Sfax, Tunisia

**Keywords:** Natural hazards, Ocean sciences, Solid Earth sciences

## Abstract

New field investigations along the East Tunisian coastline reveal sedimentary deposits and damaged localities that may account for a catastrophic event during the late Holocene. North of Sfax city, ~ 3.4 m high cliff coastal marine and alluvial terraces show 20 to 50-cm-thick chaotic layer with sandy coarse gravels mixed with reworked pebbles, broken shells of gastropods and molluscs, organic matter and Roman pottery. The chaotic layer truncates sandy-silty paleosol, covers Roman settlements and is overlain by fire remains, a thin sandy-silty aeolian unit and ~ 1-m-thick alluvial deposits. Charcoal samples collected at 10 cm below and 4 cm above the catastrophic deposits provide radiocarbon dating that brackets the catastrophic unit between 286 and 370 CE. Other historical investigations on the Roman sites of Neapolis (Nabeul), Hadrumete (Sousse), Thyna (Sfax), Meninx in Girba (Djerba), Wadi Ennouili (Gulf of Gabes), and Sabratha (in Libya) evidenced major damage and abandonment of sites in the fourth century (16, 41, 42, 43, 44). The new identification of catastrophic deposits, offshore-onshore correlations with turbidites and modelling of tsunami waves suggest the relationship with the 21 July 365 tsunamigenic earthquake (Mw ~ 8) of west Crete (Greece) and call for a better estimate of tsunami risk on the Mediterranean coastlines.

## Introduction

**Figure 1:**
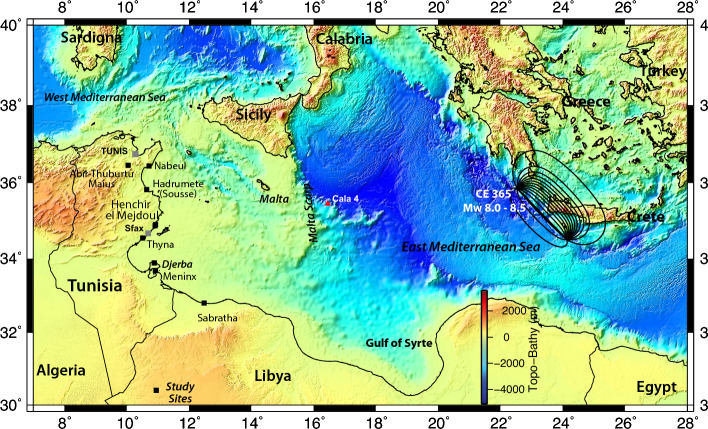
Study sites (black box) for paleotsunami record along the Tunisia coastline. The 21 July 365 Crete earthquake (Mw 8–8.5; Refs.^6,7, 51, 61^) is responsible for a major tsunami that affected the North Africa coastlines, southern European and east Mediterranean coastlines. Background topography is from GEBCO^63^. Local geological background and stratigraphic details of the paleotsunami study site are shown in Fig. 2, and Supplementary Material (SM) Figs. SM1 and SM2. The CE 365 historical earthquake generated ~ 9 m uplift in east Crete Island (Greece) and the dislocation model^61^ corresponds to a Mw 8.0–8.5 earthquake with co-seismic vertical displacement field at the surface (black contours, see also fault rupture parameters in Table 1). The study sites in coastal Tunisia face the Crete seismic source with no significant obstacle (except Malta Island) to the tsunami wave propagation. A 2.6 m thick turbidite deposits is part of the core log CALA4 (red triangle) of Fig. 4. Main archaeological sites (black boxes) in coastal Tunisia correspond to damaged and abandoned Roman cities in the fourth century (CE). Archaeological cities of Neapolis (now Nabeul), Hadrumete (now Sousse)^18,19^, Thyna (south of Sfax) and Meninx (now Djerba) in Tunisia and Sabratha in Libya show visible tsunami damage. This figure is first prepared using .ai format (Adobe Illustrator for CNRS—Delegation Alsace) 2023 Adobe Systems Incorporated and its licensors http://www.adobe.com/go/thirdparty_fr. This figure is also prepared using the Generic Mapping Tools (GMT version 5) cited in reference^62^.

The eastern Mediterranean has been the subject of several tsunamis related to catastrophic earthquakes in the past. Among the most destructive earthquakes are the Galilee earthquake in 363^1–3^, the Crete earthquake in 365^4–7^, the 1303 Rhodes Island earthquake^2,8, 9^. In central Mediterranean Sea, two major tsunamis occurred due to the 1693 earthquake offshore eastern Sicily^10^, and the 1908 Messina earthquake^11–14^. The record of the 365 Tsunami event west of Alexandria (northern Egypt) is evidenced in historical documents^2,3^, and in coastal deposits^15^. The record of paleotsunami have never been evidenced along the coastline of Tunisia. However, at Nabeul city (formerly Neapolis) in Tunisia, archaeological investigations suggest the occurrence of a catastrophic event that inundated the city after a landslide in the fourth century^16^. Near Tunisia, in Libya, records of coastal damage located in the archaeological remains of the city of Sabratha was attributed to the tsunami of 21 July 365 CE (Fig. 1; Ref.^17^). Despite the occurrence of tsunamigenic earthquakes in eastern Mediterranean, the study of geological record and impact of tsunamis on the Tunisian coastline is missing.

**Figure 2:**
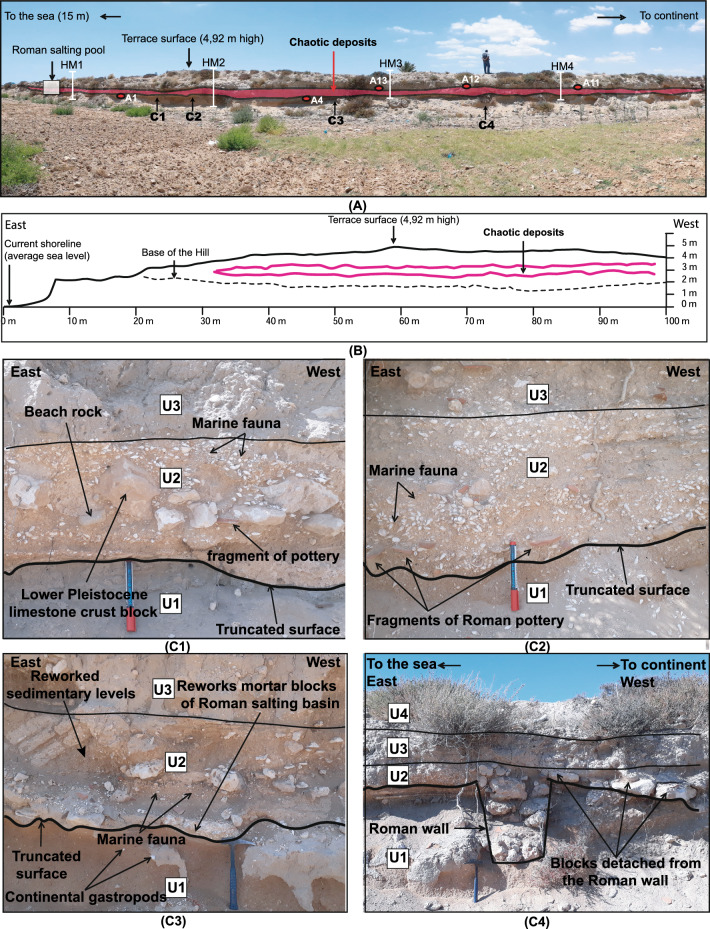
(**A**) The site at Henchir El Mejdoul (landscape photo; see location in Fig. 1). The stratigraphical cut is man-made (border of an agricultural field) and shows the catastrophic layer within the coastal late Quaternary terrace at 4.92 m above sea level (asl). The coordinates of the transect of Fig. 2 are (east point) Longitude 10.921612°, Latitude 34.903753°, (west point) Longitude 10.913605°, Latitude 34.907010°. A1, A4, A11, A12, A13 are collected samples for dating. HM 1 to 4 refer to the stratigraphic logs of Fig. 3. (**B**) East–West (left to right) total station levelling profiles made across the coastal terrace. the pink line represents the upper and lower limit of the chaotic layer, and the dashed line the base of terrace cliff outcrop (0 = present-day sea level); (**C1**–**C4**) photographs are details of the exposed chaotic layer U2 within the stratigraphic succession (with mixed beachrock, Roman pottery, Roman masonry stones with mortar, gravel, and fine sand with broken shells; see also Fig. 3); (**C4**) shows a buried Roman wall below U2. Some remaining wall bricks (similar to foundation bricks) appear as dragged in the tsunami deposits.

The ~ 650 km long east Tunisia coastline is mostly made of late Pleistocene and Holocene terraces with marine and alluvial deposits (Fig. 2; Ref.^20^). Marine deposits with Strombus Bubonius (Gastropoda Persististrombus latus), key indicator of the Marine Isotope Stage (MIS) 5e in the Mediterranean Sea, are described at different elevations above sea level, i.e. at 7 to 13 m in Cap Bon^20–26^ and 13 to 32 m in the coastal area of Monastir^25^. Further southeast in the Gulf of Gabes and the Libyan border, Holocene marine terraces show a minimum 1.9 ± 0.10 m emergence above present-day sea level since about 6000 years ago^26^. These active phenomena are either correlated with a phase of stripping and erosion of soils on the slopes, or the coastal elevation as the result of the shoreline emergence of post-glacial hydro-isostatic effects^22,27^. The relatively low topography made of marine and alluvial terraces ranging between about 1.9 m and 32 m high (above sea level) favour sea wave inundations along east Tunisia. In addition, at the end of antiquity, coastal east Tunisia recorded phenomena of silted-up land and siltation that imply a severe sudden marine inundation^27–29^.

In this paper, we present the geological context of the coastal alluvial and marine terrace deposits, followed by the stratigraphic and sedimentological sequence of the chaotic deposits dated fourth century in the eastern Tunisian coastline. A search of historical documents and archives on the coastal and archaeological sites allows us to highlight the record of major damage during the fourth century CE. A turbidite deposit record collected east of Malta Island shows the correlation with the 365 CE Crete earthquake. Numerical modelling of tsunamis with the seismic source on the western Crete and Hellenic subduction zone help to validate the hypothesis of wave propagation generated by a major tsunamigenic earthquake in the Eastern Mediterranean. We then discuss the implications of the occurrence of a large tsunamigenic earthquake event on the North Africa and eastern Mediterranean coastlines.

## Other geological evidence of paleotsunami records

Geological records of tsunamis generated by major subduction zone earthquakes have been identified and characterized in several regions worldwide^30^. Tsunami deposits inland can be distinguished from other deposits by their relatively thick (10 to 70 cm) chaotic sediments made of gravel in a silty-sand matrix mixed with broken shells and with no layering structure. The succession of intertidal fine sediments with flooded and buried forests probably related to six paleotsunami events during the last 7000 years is dug in the western United States^31^. Following the Sumatra tsunamigenic earthquake (2004, Mw 9.1), stratigraphic studies and geochemical analysis with isotopic dating of trench and core deposits of the Andaman Island reveal the occurrence of a minimum of seven tsunami events during the past 8000 years^32^.

In southern Sicily, sedimentological study and benthic foraminifera analysis aided by X-ray fluorescence data from cores collected in the northern part of Augusta Bay, allowed the identification of horizons related to three historical tsunamis correlated with the Current Era (CE), namely the 1542, 1693 and 1908 large earthquakes^33,34^.

In Tunisia, Di Vita^35,36^ refers to several Roman inscriptions dated to 368–370 CE, and among them that of Abbir-Thuburbo Maius on building retrofitting and sea-wave damage (Fig. 1). North of Sfax city, Khadraoui et al.^37^ infer a ~ 250 years cal. BP paleotsunami event from the study of lagoonal units in cores with coarse marine sands and shelly bed covered by deposits with charcoal particles and Roman pottery fragments. In coastal Cap Bon area near Tunis, May et al.^38^ show evidence of imbricated boulders accumulation, transported ~ 50 m inland and suggest a correlation with either an extreme storm event or a tsunami.

West of Alexandria coastline in northern Egypt, macrofossil determination, XRD analysis, magnetic susceptibility, particle size analysis, total organic and inorganic matter measurements, and radiocarbon dating of deposits in trenches and cores show a correlation with tsunamigenic earthquakes occurred along the Hellenic subduction zone in 1600 BCE and CE 365, 1303, and 1870^3,14^. Furthermore, using geophysical surveys and radiocarbon dating of sediment cores collected east of the Malta escarpment in the Ionian Sea, Polonia et al.^39^ identify a thick (up to 20–25 m) megaturbidite contemporaneous with the CE 365 Crete earthquake.

## Results

### Geological and sedimentary analysis

Field investigations near the Roman site of Henchir El Majdoul located ~ 22 km north of Sfax city expose a ~ 4 m-high cliff outcrop with four distinct sedimentary units of coastal marine and alluvial deposits (Figs. 1 and 2A).

**Figure 3:**
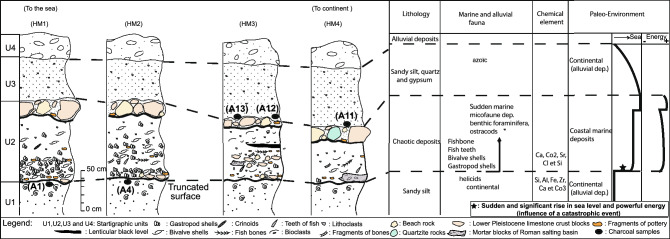
Stratigraphic log of four sections of outcrops from the mixed marine and alluvial terrace at Henchir El Mejdoul (see location in Fig. 2A). A 40 to 100 cm-thick chaotic layer is characterized by an erosion basal limit and mixed content of broken shells, pebbles, Roman masonry stones and beachrock. The chemical analysis is from Khadraoui et al.^26^, and the sea level change (Paleo-environment) is from Paskoff and Salanville^21^ and Lambeck et al.^23^. Radiocarbon dating of 5 charcoal samples that bracket the chaotic—catastrophic layer (see Table SM1 in supplementary material).

The survey of topographic profiles by means of differential GPS across the coastal terrace provides the elevations of the four units (Fig. 2B). From bottom to top, we distinguish a stratigraphic succession with units U1, U2, U3 overlain by recent soil U4 (Figs. 2 and 3, and Fig. SM2). We observe:

- A 0.2 m to 0.5 m thick unit U2 made of broken shells, beachrock, fine marine sand mixed with alluvial sandy-gravel, Roman pottery, Roman masonry stones. abundant molluscs shells (gastropods and bivalves).
- The chaotic U2 layer shows an erosion base limit that truncates U1 made of fine sand with Helix.
- Fine sand with alluvial gravel Unit U3 covers the chaotic layer.
- Radiocarbon dating of five charcoal samples collected immediately below and above the chaotic layer (Table SM1) and related Oxcal Bayesian simulation (Fig. SM3) provide an age bracket between 286 and 370 cal. CE which correlates with the 365 CE large earthquake tsunami of western Crete.
- The tsunami wave propagation may generate turbidite—homogenite in between Malta and Tunisia.

The transition between the mixed deposits U2 and the underlying sedimentary unit U1 is abrupt and represented by an undulating erosion surface (Figs. 2B,C and 3). U2 near the coastline becomes less fossiliferous and richer in clasts and locally reworked mortar blocks towards the mainland (Fig. 3).

In uppermost U2 mixed deposits, centimetric to decimetric blocks are made of either the remains of wall stones of quartzite or beach rocks made of calcarenite fragments (Figs. 2C and 3). Before reaching the continent, U2 also shows the same species of molluscs with an increase of fish bones and teeth, bone fragments and crinoids. This fauna becomes relatively less abundant and more diverse.

Laterally, the marine fauna is represented essentially by the debris of shells of molluscs. In addition, the high concentrations of the marine geochemical proxy elements such as Sr, Ca and Cl (obtained from spectrometer measurements^26^, in the chaotic facies characterize a marine environment and suggest a biogenic origin. At the top of U2, we note the presence of a lenticular centimetric level of black silts rich in charcoal that contains some lithoclasts, pottery fragments and bioclasts (Fig. 3). Unit U3 (100 cm), is deposited directly above the erosional contact on the unit U2 and comprise sandy silt rich in centimetric to decimetric blocks made of wall stones of quartzite and calcarenite fragments characterizing a continental origin (Figs. 2C and 3). Unit U4 (30 cm) corresponds to alluvial deposits.

The Roman pottery artefacts in U2 consists in different items with a lamp, a dish and a bowl, and an amphora mostly indicating a period between the first and fourth century CE (see Fig. SM4 and related text in Supplementary Material). Accelero-mass spectrometry (AMS) radiocarbon analysis of two charcoal samples (A1 and A2) collected at ~ 0.10 m below Unit U2 at top of Unit U1 provides the following 2σ (94.5%) calibrated CE (current era) ages: A1 = 80–230 and A4 = 236–385, while three charcoal samples A11, A12, and A13 collected at ~ 0.02 m above U2 at the base of Unit U3) show the following 2σ (94.5%) calibrated CE: A11 = 295–577; A12 = 252–556 and A13 = 249–541 (Table SM1 and Fig. SM3).

The stratigraphy of the mixed deposits (U2) of Henchir El Majdoul with several blocks not laid flat shows a chaotic deposition. The abundance of a diversified marine fauna (molluscs, foraminifera, ostracods, see Fig. 3), the reworked pebbles and boulders in U2 and truncated U1 surface, indicate a transport and a sedimentation caused by the influence of a violent marine current (runup wave) that invaded the continental part of the back beach (Figs. 2C and 3) toward the period 286–370 CE (2σ; see Fig. SM3) that brackets U2. Offshore records of high energy and megaturbidites are also observed over a wide region offshore east of Tunisia and the Eastern Mediterranean Sea, and between the tectonically active Calabria and Hellenic subduction zones^39,40^. Sedimentological reconstructions with dating suggest that the tsunami following the CE 365 Crete earthquake produced giant turbidity currents along a front over 2000 km long, and particularly along the Malta escarpment from northern Africa to Italy (Fig. 1).

### Archeological and historical evidence of earthquake damage

The search of documents and archives on the archaeological sites and their history highlights the damage and the decline of several sites and localities during the fourth century along the eastern Tunisian coast (Fig. 1). From the end of the Punic to the Roman period, several authors report the occurrence of significant damage in archaeological sites during the fourth century. Meninx Island (now Djerba, Fig. 1), the largest Roman town of the Tunisian coast, adopted the new name Girba (from Antonin's sailing itinerary book and Latin geographer, according to Julius Honorius Orator in his Cosmographia) following inundations and damage in the second half of the fourth century^41,42^. In the Gulf of Gabes, field investigations highlight the occurrence of a rhexistasis phase (stripping and entrainment of soils, erosion of soft rocks on the slopes by runoff and colluvium, and correlative impasto of the lower parts of the topography by the mobilized materials) dated at the end of the Roman period^43,44^. A particularly revealing site in this respect is the Henchir Chaabane (33.36° N; 11.13° E) where an ancient Roman quarry is being inundated and cleared by the sea from the detrital cover at the fourth century. Close to Djerba (Fig. 1), in Wadi Ogla (33.56° N, 11.09° E) and in the neighbouring river Wadi Ennouili, a 4 m thick terrace contains abundant pottery shards dated to the 4th or fifth century CE as markers of extensive damage (Fig. 1; Ref.^44^). The same phenomenon is found towards the northern end of the Gulf of Gabès, in the islet of Djezira (35.1678° N; 11.0791° E) where a significant layer of colluvium also packs shards of Roman pottery. South of the city of Sfax, extensive archaeological studies describe the coastal city of Thyna (now Henchir Thyna; Fig. 1) that was a blooming town during second and third century CE and underwent severe damage and restoration that shows new houses (with double wall design in Fig. SM5, and damaged bath in Fig. SM6) with western migration (away from the sea) in the fourth century^44,45^. All along the coast of Sfax and near the current shoreline, most of Roman constructions have been destroyed, while all the Byzantine constructions (such as Ksar Younga at 30 km south of Sfax) remain intact.

Our field observations show evidence of restoration work of buildings in excavations of Thyna city dated using pottery fragments and archaeological artefacts of the fourth century (Fig. SM4 in Supplementary Material). To the north, at Hadrumète (now Sousse, Fig. 1) which was until the third century CE an extensive and prosperous city with an important harbour, only the two moles remain (one to the north near the Quarantine and the other to the south) as well as sections of an intermediate breakwater equipped with vents to dampen the strong waves. According to the Anonymi Stadiasmus Maris Magni^41^, the city was abandoned due to the silted port and related economic problems at the end of 4th or at the beginning of fifth century CE. According to Kamoun et al.^29^, Acholla city (located along the coastline about 45 km north of Sfax city) was also abandoned toward the 4th to fifth century after the construction of a pier, which deflected longshore drift currents, accelerated the leeward progradation of the sandspit sediments and finally led to the leeward silting of the coastal harbour habitats (Fig. SM5). Further north in the fourth century, Neapolis (now Nabeul, Fig. 1) was partially submerged and damaged by a catastrophic wave and experienced an important landslide^16^; the new set of observations and archeological dating correlates the catastrophic event of Neapolis to the tsunami of CE 365 (Ref.^16^; Fantar, personal communication).

### Turbidite deposits offshore Tunisia contemporaneous of the 365 CE tsunami

The marine record that has the potential of reconstructing a more complete record of high-energy sedimentary events triggered by seismic activity. Indeed, the occurrence of deep sea megaturbidites deposited during the CE 365 event in east Mediterranean and near Malta Island suggest that the tsunami wave produced multiple far-field slope failures that resulted in stacked basal turbidites^46^.

The CE 365 tsunamigenic earthquake occurred in west Crete may have crossed the Mediterranean Sea to generate megaturbidites near the Malta escarpment and chaotic deposits on the Tunisian coast (Fig. 1).

**Figure 4:**
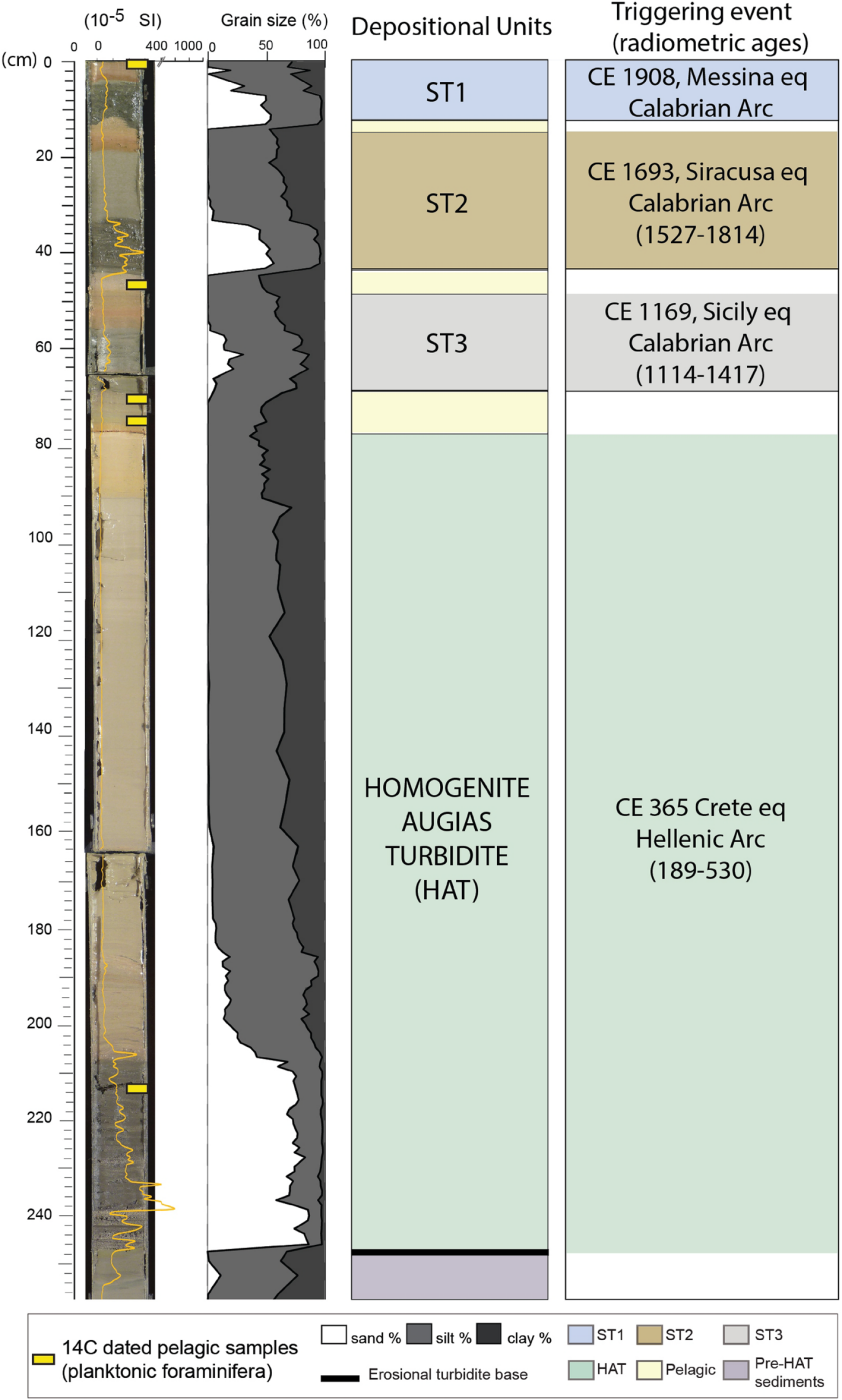
Log of core *CALA-04. From left to right:* photograph with high-resolution magnetic susceptibility in yellow, grain size as %, subdivision in individual ST and pelagic units identified by different colours, triggering events and radiometric ages for each seismo-turbidite. See Fig. 1 for core location and Table SM2 for turbidite descriptions and dating.

The gravity core CALA-04 collected in the western Ionian Sea, close to the Malta escarpment (Fig. 1) reveals that turbidites and megaturbidites are prevalent in the flat deep basin floor where pelagites deposited during interseismic periods represent less than 10% of the total sediment emplaced during the last 2 kyrs (yellow layers in Fig. 4). Below the three Calabrian Arc seismo-turbidites (ST1, ST2 and ST3 in Fig. 4), a megabed known in the literature as Homogenite^47^, Augias turbidite^48^ or HAT (Homogenite/Augias turbidite)^49^ has peculiar sedimentological and compositional characters that make it unique in the historical sedimentary record. In core CALA-04 (Fig. 4) it is about 1, 70 m thick, but it can reach up to 20–25 m in open abyssal plains or in confined deep basins close to the Africa coast^46,49^. It is characterized by a sharp increase in sand content represented by a heterogeneous mixture of detrital mineral grains and biogenic components from different sources, whose mineralogy can be traced back to southern Italy, Malta and Africa margins^50^. ^14^C dating in this core provide an emplacement time window of CE 189-530 (Table SM1). A Calabrian Arc earthquake is unlikely to have triggered the deposition of such megabed because no major earthquakes are described in the catalogues during this time window. Moreover, average magnitude earthquakes from the Calabrian Arc usually produce the deposition of decimetric seismo-turbidites but not megabeds^47^.

The observed and cited damage in Table SM3 and the disappearance of several sites in coastal areas extending along more than 800 km from the city of Sabratha in Libya to the city of Neapolis in northern Tunisia suggest the occurrence of high amplitude waves (> 4 m) and extending for a minimum of 500 m westward inland (according to the altitude and outcrop of the chaotic deposit U2 at Henchir El Majdoul, respectively; see Figs. 2A,B and SM1). Such a catastrophic event should result from a high magnitude tsunamigenic earthquake in the eastern Mediterranean. In addition, archive documents^6,13, 14^ also show that the most significant tsunamigenic earthquake capable of affecting east Tunisia coast of is that of the Hellenic subduction zone of July 21, 365 with Mw ~ 8.0–8.5^7,9, 28^. To validate this hypothesis, numerical modelling with a seismic source and rupture located west of Crete (Greece) has been carried out to explain the generation of large tsunami waves that can affect the eastern coast of Tunisia, as reported in the next Section.

## Modelling

Large earthquakes associated with major tsunami waves occurred in 365, 1303 and 1870 along the western, central and eastern Hellenic subduction zone, respectively, have been reported by several authors^2,3^. The CE 365 major earthquake affected the whole east Mediterranean coastlines. We here use for tsunami numerical modelling the parameters of an Mw 8 earthquake, consistent with the rheological and dynamic properties of the tectonic structures and subduction zone (Ref.^7^; Fig. 1).

The geometric and kinematic properties of the fault zone are inferred mainly from the surface deformation and 7–9 m uplift measured along the coastline west of Crete and from damage distribution^2,6, 7^. We also use the scenario of an earthquake of similar moment magnitude occurring at approximately the same location^51^. This is possibly inspired but not directly meant to reproduce the 365 historical event. The fault parameters of the two selected scenarios are reported in Table 1. We used uniform slip on planar faults embedded in a homogeneous elastic half-space to calculate the initial instantaneous seafloor displacement (Ref.^51^; see for example Fig. 1 for the displacement corresponding to the fault model of 7).

**Table 1:** Source parameters used for tsunami numerical modelling and maximum modelled height over the simulation domain (see “Modelling” section).

Source	Longitude	Latitude	Length (km)	Width (km)	Depth (km)	Slip (m)	Strike (deg)	Dip (deg)	Rake (deg)	Max. height (m)
(1) Lorito et al.^54^	22.41665 W	36.00025 N	130	86	24,664	17.5	314	35	90	~ 22
(2) Shaw et al.^7^	23.4 W	35.2 N	100	90	22.5	20	315	30	90	~ 33

Coordinates and depth refer to the geometrical centre of the fault.

**Figure 5:**
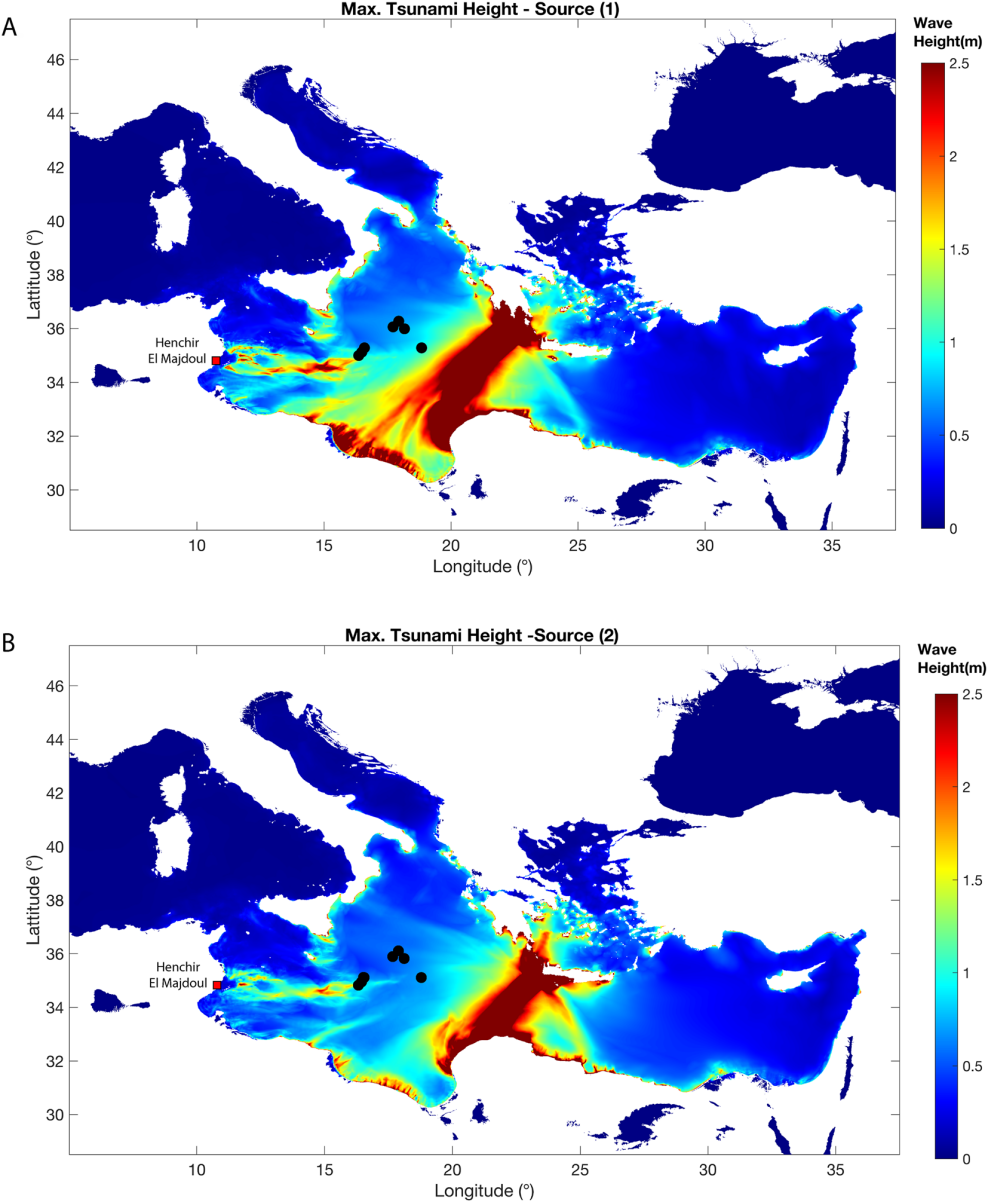
Distribution of Max. Tsunami Wave Height for source 1 (**A**)^29^ and source 2 (**B**)^7^. Fault parameters for modelling are in Table 1. This figure is prepared using MATLAB version: 9.7.0 (R2019b), Natick, Massachusetts: The MathWorks Inc. https://www.mathworks.com).

**Figure 6:**
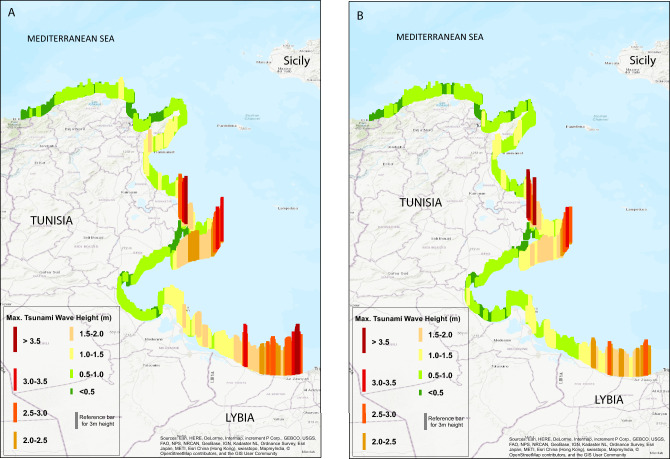
(**A**,**B**) Maximum tsunami wave height on the Tunisia-Libya coastline as a result of the simulation for Source-1 ((**A**); Ref.^51^) and Source-2 ((**B**); Ref.^7^) on 5 m isobath extrapolated at 1 m with the Green’s law. Both simulations of maximum wave heights agree with the observed outcrop (Fig. 2A) and estimated minimum 500 m chaotic layer (inundation area) west of the coastline at Henchir El Majdoul (see also the additional map Fig. SM1 in Supplementary Material). This figure is prepared using ArcGIS (ESRI, R. (2011)). ArcGIS desktop: release 10. Environmental Systems Research Institute, CA, 634, 315–325. https://www.esri.com/en-us/home).

Tsunami numerical modelling is performed using the nonlinear shallow water equations implemented in spherical coordinates in the Tsunami-HySEA finite volumes numerical code^52,53^) The SRTM30 + topography-bathymetry data are used (https://topex.ucsd.edu/WWW_html/srtm30_plus.html) with a 30 arc-sec resolution grid in the domain 5° E–37° E and 28.5° N-47.5° N. The simulation duration is set as 8 h for both sources. The modelled tsunamis affect the entire Central and Eastern Mediterranean Sea. Maximum tsunami wave and water energy fields modelled from both fault sources reach predominantly the Libyan Cyrenaica coastline, Gulf of Syrte and Gulf of Gabes in eastern Tunisia (Fig. 5), with slightly different patterns and intensities due to the differences in the source parameters. The maximum tsunami height values peak at ~ 22 and ~ 33 m in the near field, respectively for Lorito et al.^54^ and for Shaw et al.^7^ seismic sources. The simulation of maximum wave amplitude at this resolution is not suitable for detailed run-up modelling. To get an idea of the potential run-up for this type of sources, the values in eastern Tunisia (Fig. 6), picked at the 5 m isobath, are extrapolated at 1 m depth with the Green’s law^55–57^. The modelled values exceed 3 m at several locations along the coast of Sfax region.

## Discussion and conclusion

The study of marine and alluvial terrace at Henchir site along the shoreline in southern Tunisia (north of Sfax city) shows evidence of a chaotic catastrophic layer that may be correlated with a past tsunami event. The stratigraphic, sedimentological, archaeological and isotopic dating evidence converges towards the identification of a catastrophic event in the fourth century along the east Tunisian coast.

The correlation between the chaotic unit U2 as described immediately north of Sfax city in Henchir El Mejdoul site and the CE 365 tsunamigenic earthquake is attested by the mixed marine and alluvial sedimentary content of U2 unit and made of mixed marine and alluvial deposits, Roman archaeological artefacts made of fragments of walls and pottery shards. The radiocarbon dating of charcoal samples that provides an age range of CE 236-541 and age estimate of Roman potteries are all consistent with the occurrence of a catastrophic event during the fourth century CE. Indeed, in addition of the stratigraphic evidence of catastrophic layer, the correlation between reports on damage and inundations in several archaeological cities from the northern Neapolis site (presently Nabeul) in eastern Tunisia to the southern Sabratha site in NW coastal Libya attests for the occurrence of a catastrophic event and tsunami wave during the fourth century CE. According to the historical earthquake catalogues and recent seismotectonic studies, no local major historical earthquake of the fourth century can account for such coastal damage in Tunisia^58,59^. The direct correspondence between the U2 deposit alone and the CE 365 tsunamigenic earthquake can be debated but the coincidence with the damage of numerous archaeological sites and economic collapse of Roman settlements supports the correlation with an earthquake capable of producing devastating effects over ~ 800 km long coastline (eastern Tunisia and northern Libya) which requires a large magnitude event that can only be ascribed to the Mediterranean subduction zones. In the Calabrian Arc no similar events are known in this time interval, while in the Hellenic Arc the CE 365 earthquake and tsunami are known to have produced basin-wide effects as far as Albania, southern Italy, and northern Africa along a front over 2000 km long.

The Hellenic subduction zone is the main source for the occurrence of a major earthquake tsunami in the east Mediterranean area. The NW–SE trending, NE dipping subduction zone in west Crete, and related earthquake rupture parameters has the potential for the generation of a destructive wave propagation that may affect the Tunisian – Libyan coastline (Fig. 6). Other earthquakes related to historical tsunamis in 1908, 1693, and 1169 affected southern Italy and among them the two latter only probably apply to the possible propagation to the Tunisia coastline^54^. However, the detailed studies of earthquake rupture sources with pure thrust mechanism on the ENE-WSW striking and NW dipping Calabria subduction zone, and related modelling of tsunami wave propagation imply 1.3 m uplift to the SE, to 0.6 m subsidence and predict 1–3 m high wave amplitudes in Sicily and neighbouring coastlines^50,60^. Although significant sea waves and submarine landslides were reported in this region, these historical earthquakes did not generate major tsunami waves with seafloor displacement and seismic energy that would affect North African and all the eastern Mediterranean coastlines.

Radiometric ages in an area of the Eastern Mediterranean Sea greater than 150,000 km^2^ provide evidence of a single basin-wide event within the time window CE 364–415^54^ that can be correlated with a catastrophic tsunami sourced from the CE 365 Crete megathrust earthquake that produced devastating effects including widespread massive sediment remobilization in the Eastern Mediterranean Sea. Seismic shaking from the Crete earthquake was probably unable to trigger mass movements 600–800 km from the epicentre where the HAT was sampled. Sedimentological and compositional reconstructions suggest that the tsunami following the Crete earthquake produced giant turbidity currents along a front over 2000 km long, from northern Africa to Italy. When the tsunami wave hit the steep Malta escarpment, it propagated on the shallow and wide continental shelf of the Tunisia-Sicily Channel as well as on the shelf of the Sirte and Gabes Gulf. The water back-surge eroded extensively the continental shelves triggering gigantic turbidity currents that transported shallow water detritus to the abyssal plain^60^.

The numerical modelling of tsunami wave propagation using 2 different seismic sources for the 365 major earthquake shows up to 4-m-high wave height that affect the coastal region of Tunisia. The height of the chaotic U2 deposit reaching 3 to 3.5 m high at Henchir El Majdoul site indicates the likely minimum wave height that overflowed the Tunisian coastline in CE 365. A recent assessment for the region points to a significant tsunami hazard on the coasts of Tunisia due to earthquake sources in the Mediterranean^53^. However, this is a region-wide model that has not addressed the details of the inundation probability with the high resolution of a local study (e.g. Ref.^61^). These results call for the development of a detailed local tsunami hazard assessment in coastal Tunisia.

### Supplementary Information

Supplementary Information.

## Data Availability

All data presented in this article can be obtained on request to the first author Dr. Nejib Bahrouni bahrouni_nejib@yahoo.fr .

## References

[1] Kenneth W, Russell KW (1980). The earthquake of May 19, A.D. 363. Am. Sch. Orient. Res..

[2] Guidoboni E, Comastri A, Traina G (1994). Catalogue of Ancient Earthquakes in the Mediterranean Area up to the 10th Century.

[3] Ambraseys NN (2009). Earthquakes in the Mediterranean and Middle East: A Multidisciplinary Study of Seismicity up to 1900.

[4] François J, Bernard B (1984). Le raz de marée du 21 juillet 365. Mélanges de l’École Française de Rome, Antiquité, Tome.

[5] Lepelley C (1984). L'Afrique du Nord et le prétendu séisme universel du 21 juillet 365. Mélanges de l'École française de Rome Antiquité.

[6] Stiros S (2001). The AD 365 Crete earthquake and possible seismic clustering during the fourth to sixth centuries AD in the Eastern Mediterranean: A review of historical and archaeological data. J. Struct. Geol..

[7] Shaw B, Ambraseys NN, England PC, Floyd MA, Gorman GJ, Higham TFG, Jackson JA, Nocquet J-M, Pain CC, Piggott MD (2008). Eastern Mediterranean tectonics and tsunami hazard inferred from the AD 365 earthquake. Nat. Geosci..

[8] Papazachos BC (1996). Large seismic faults in the Hellenic arc. Ann. Geofis..

[9] Papadopolous GA, Daskalaki E, Fokaefs A, Giraleas N (2007). Tsunami hazards in the Eastern Mediterranean: Strong earthquakes and tsunamis in the East Hellenic Arc and Trench system. Nat. Hazards Earth Syst. Sci..

[10] Piatanesi A, Tinti S (1998). A revision of the 1693 eastern Sicily earthquake and tsunami. J. Geophys. Res. Solid Earth.

[11] Baratta M. La catastrophe du tremblement de terre de Calabre et Messine (28 Décembre 1908), 2 volumes, Rome, Société géographique italienne, 1909, OCLC 38646170 (1909).

[12] Hobbs W. H. Le tremblement de terre de Messine, American Geographical Society, 1909, OCLC 25786357 (1909).

[13] Mauritius I (1910). Résultats d'une enquête sur le tremblement de terre de Messine. Ann. Géogr..

[14] Guidoboni E, Ferrari G, Tarabusi G, Sgattoni G, Comastri A, Mariotti D, Valensise G (2019). CFTI5Med, the new release of the catalogue of strong earthquakes in Italy and in the Mediterranean area. Sci. Data.

[15] Salama A, Meghraoui M, El Gabry M, Maouche S, Hussein MH, Korrat I (2018). Paleotsunami deposits along the coast of Egypt correlate with historical earthquake records of eastern Mediterranean. Nat. Hazards Earth Syst. Sci..

[16] Fantar M, Spanu PG, Zucc R (2019). « Un decennio di esplorazioni nella colonia Iulia Neapolis in Africa Proconsolare », Atti dell’Accademia Nazionale dei Lincei, anno CDXV, Rendiconti se, rie IX-Volume XXIX- Fascicolo 3–4. Roma.

[17] Di Vita A., Sismi, urbanistica e cronologia assoluta. Terremoti e urbanistica nelle città di Tripolitania fra il I secolo A.C. ed il IV D.C. In: L'Afrique dans l'Occident romain (Ier siècle av. J.-C. - IVe siècle ap. J.-C.) Actes du colloque de Rome (3–5 décembre 1987), 425–494 (École Française de Rome, 1990).

[18] Foucher, L. Hadrumetum. Paris, PUF, 1964; one vol. in-8°, 408pp. Publication of the University of Tunis, Faculty of Letters, 1st Series, vol. X (1964).

[19] Laporte, J. P. D'Hadrumète à Sousse, des années 350 à 859. RM2E, Revue de la mediterranée, Ed. Electronique, Tome II-1, 3–34 (in Corsican) (2015).

[20] Bouaziz S, Jedoui Y, Barrier E, Angelier J (2003). Neotectonics in the Tyrrhenian marine deposits of the southeastern Tunisian coast: Implications for sea level changes. C. R. Geosci..

[21] Paskoff, R., Salanville, P. Les côtes de la Tunisie: Variation du niveau marin depuis le Tyrrhénien, Éditions Maison de l’Orient, 192. (1983).

[22] Jedoui Y, Kallel N, Michel F, Ismail HB, M’Rabet A, Montacer M (1998). A high relative sea-level stand in the middle Holocene of southeastern Tunisia. Mar. Geol..

[23] Lambeck K, Yokoyama Y, Purcell T (2002). Into and out of the last glacial maximum: Sea-level change during oxygen isotope stages 3 and 2. Quat. Sci. Rev..

[24] Elmejdoub N, Jedoui Y (2009). Pleistocene raised marine deposits of the Cap Bon peninsula (N–E Tunisia): Records of sea-level highstands, climatic changes and coastal uplift. Geomorphology.

[25] Mahmoudi M (1988). Nouvelle proposition de subdivisions stratigraphiques des dépôts attribués au Tyrrhénien en Tunisie (Région de Monastir). Bull. Soc. Géol. France.

[26] Morhange C, Pirazzoli PA (2005). Mid-Holocene emergence of southern Tunisian coasts. Mar. Geol..

[27] Amrouni O, Hzami A, Heggy E (2019). Photogrammetric assessment of shoreline retreat in North Africa: Anthropogenic and natural drivers. ISPRS J. Photogram. Remote Sens..

[28] Oueslati A (1995). The evolution of low tunisian coasts in historical times: From proradation to erosion and salinization. Quat. Int..

[29] Kamoun M, Zaïbi C, Langer MR, Khadraoui A, Ben Hamad A, Ben Khalifa Kh, Carbonel P, Ben Youssef M (2020). Environmental evolution of the Acholla coast (Gulf of Gabes, Tunisia) during the past 2000 years as inferred from paleontological and sedimentological proxies. Neues Jahrb. Geol. Paläontol..

[30] McCalpin JP (1996). Paleoseismology.

[31] Atwater BF (1987). Evidence for great holocene earthquakes along the outer coast of Washington State. Am. Assoc. Adv. Sci. New Ser..

[32] Malik JN, Johnson FC, Khan A, Sahoo S, Irshad R, Paul D, Arora S, Baghel PK, Chopra S (2019). Tsunami records of the last 8000 years in the Andaman Island, India, from mega and large earthquakes: Insights on recurrence interval. Nat. Res. Sci. Rep..

[33] De Martini PM, Barbano MS, Smedile A, Gerardi F, Pantosti D (2010). A unique 4000 year-long geological record of multiple tsunami inundations in the Augusta Bay (Eastern Sicily, Italy). Mar. Geol..

[34] Smedile A, Molisso F, Chagué C, Iorio M, De Martini PM, Pinzi S, Collins PEF, Sagnotti L, Pantosti D (2019). New coring study in Augusta Bay expands understanding of offshore tsunami deposits (Eastern Sicily, Italy). Sedimentology.

[35] Di Vita A (1980). Evidenza dei terremoti del 306–310 e del 365 D.C. in Tunisia. Antiq. Afr..

[36] Di Vita A (1995). Archeologists and earthquakes: The case of 365 AD. Ann. Geofis..

[37] Khadraoui A, Kamoun M, Ben Hamad A, Zaïbi C, Bonnin J, Viehberg F, Bahrouni N, Sghari A, Abida H, Kamoun F (2018). New insights from microfauna associations characterizing palaeoenvironments, sea level fluctuations and a tsunami event along Sfax Northern coast (Gulf of Gabes, Tunisia) during the Late Pleistocene-Holocene. J. Afr. Earth Sci..

[38] May, S. M., Willershaüser, T., Vött, A., Boulder transport by high-energy wave events at Cap Bon (NE Tunisia), Schwarzer, Schrottke and Stattegger eds, *From Brazil to Thailand – New results in Coastal Research*, Coastline Reports 16, 01–10 (2010).

[39] Polonia A, Bonatti E, Camerlenghi A, Lucchi RG, Panieri G, Gasperini L (2013). Mediterranean megaturbidite triggered by the AD 365 Crete earthquake and tsunami. Nat. Sci. Rep..

[40] Polonia A, Vaiani CS, de Lange GJ (2016). Did the AD 365 Crete earthquake/tsunami trigger synchronous giant turbidity currents in the Mediterranean Sea?. Geology.

[41] Magni ASM (2010). Geographi Graeci minores (Cambridge Library Collection – Classics).

[42] Drine, A. Les entrepôts de Méninx. In *Antiquités africaines*, 43, L'Afrique du Nord de la protohistoire à la conquête arabe, 239–251. doi: 10.3406/antaf.2007.1427 (2007).

[43] Beschaouch AD (1986). l'Africa latino-chrétienne à l'Ifriqiya arabo-musulmane: Questions de toponymie. Comptes Rendus des séances de l'Académie des Inscriptions et Belles-Lettres.

[44] Slim H, Trousset P, Paskoff R, Oueslati A, Bonifay M, Lenne J (2004). Le Littoral de la Tunisie: Etude Géoarchéologique et Historique.

[45] Thirion J (1957). Un ensemble thermal avec mosaïques à Thina (Tunisie). Mélanges d’archéologie et d’histoire.

[46] Polonia A, Panieri G, Gasperini L, Gasparotto G, Bellucci LG, Torelli L (2013). Turbidite paleoseismology in the Calabrian Arc Subduction Complex (Ionian Sea). Geochem. Geophys. Geosyst..

[47] Kastens KA, Cita MB (1981). Tsunami-induced sediment transport in the abyssal Mediterranean Sea. Geol. Soc. Am. Bull..

[48] Hieke W, Werner F (2000). The *Augias*
*megaturbidite* in the central Ionian Sea (central Mediterranean) and its relation to the Holocene Santorini event. Sediment. Geol..

[49] Cita MB, Rimondi B (1997). Geological and geophysical evidence for a Holocene tsunami deposit in the Eastern Mediterranean deep-sea record. J. Geodyn..

[50] Gutscher M-A, Roger J, Baptista M-A, Miranda JM, Tinti S (2006). Source of the 1693 Catania earthquake and tsunami (Southern Italy): New evidence from Tsunami modeling of a locked subduction fault plane. Geophys. Res. Lett..

[51] Stiros S (2010). The 8.5+ magnitude, AD365 earthquake in Crete: Coastal uplift, topography changes, archaeological and historical signature. Quat. Int..

[52] Macías J, Mercado A, González-Vida JM, Ortega S, Castro MJ (2016). Comparison and Computational Performance of Tsunami-HySEA and MOST Models for LANTEX 2013 Scenario: Impact Assessment on Puerto Rico Coasts.

[53] Carrier GF, Greenspan HP (1958). Water waves of finite amplitude on a sloping beach. J. Fluid Mech..

[54] Lorito S, Tiberti MM, Basili R, Piatanesi A, Valensise G (2008). Earthquake generated tsunamis in the Mediterranean Sea: Scenarios of potential threats to Southern Italy. J. Geophys. Res..

[55] Synolakis CE (1987). The run-up of solitary waves. J. Fluid Mech..

[56] Synolakis CE (1991). Green’s law and the evolution of solitary waves. Phys. Fluids A.

[57] De la Asunción M, Castro MJ, Fernández-Nieto ED, Mantas JM, Acosta SO, González-Vida JM (2013). Efficient GPU implementation of a two waves TVD-WAF method for the two-dimensional one-layer shallow water system on structured meshes. Comput. Fluids.

[58] Kharrat S, Harbi A, Meghraoui M, Bouaziz S (2018). The Tunisian homogenized macroseismic database (second century to 1981): First investigations. Seismol. Res. Lett..

[59] Bahrouni N, Bouaziz S, Soumaya A, Ben Ayed N, Attafi K, Houla Y, El Ghali A, Rebai N (2013). Neotectonic and seismotectonic investigation of seismically active regions in Tunisia: A multidisciplinary approach. J. Seismol..

[60] Gibbons SJ, Lorito S, Macías J, Løvholt F, Selva J, Volpe M, Sánchez-Linares C, Babeyko A, Brizuela B, Cirella A, Castro MJ, de la Asunción M, Lanucara P, Glimsdal S, Lorenzino MC, Nazaria M, Pizzimenti L, Romano F, Scala A, Tonini R, Manuel Gonzalez Vida J, Voge M (2020). Probabilistic Tsunami hazard analysis: High performance computing for massive scale inundation simulations. Front. Earth Sci..

[61] Basili R (2021). The making of the NEAM Tsunami hazard model 2018 (NEAMTHM18). Front. Earth Sci..

[62] Wessel P, Smith WHF, Scharroo R, Luis J, Wobbe F (2013). Generic mapping tools: Improved version released. EOS Trans. AGU.

[63] Becker JJ, Sandwell DT, Smith WHF, Braud J, Binder B, Depner J, Fabre D, Factor J, Ingalls S, Kim S-H, Ladner R, Marks K, Nelson S, Pharaoh A, Trimmer R, Von Rosenberg J, Wallace G, Weatherall P (2009). Global bathymetry and elevation data at 30 arc seconds resolution: SRTM30_PLUS. Mar. Geodesy.

