# Supplementary material for: Tsunami deposits in Tunisia contemporaneous of the large 365 CE Crete earthquake and Mediterranean Sea catastrophic event
Source: Sci Rep. 2024 Feb 24;14:4537. doi: 10.1038/s41598-024-53225-7 (PMC10894223; doi:10.1038/s41598-024-53225-7)
Supplement: Supplementary file 1 — Supplementary Information. [file 41598_2024_53225_MOESM1_ESM.docx]

SUPPLEMENTARY MATERIAL

Tsunami deposits in Tunisia contemporaneous of the Large 365 CE Crete earthquake and Mediterranean Sea

catastrophic event

Nejib Bahrouni ^1^, Mustapha Meghraoui ^2*^, Hafize Başak Bayraktar ^3^,

Stefano Lorito ^3^, Mohamed Fawzi Zagrarni ^4^, Alina Polonia ^5^,

Nabil Bel Mabrouk ^6^, and Fekri Kamoun ^7^

*^1^* Office National des Mines, Tunis, Tunisia

*^2^* ITES, CNRS-UMR 7063, Université de Strasbourg, F-67084, France

*^3^ Istituto Nazionale di Geofisica e Vulcanologia (INGV), Rome, Italy*

*^4^ Institut Supérieur des Sciences et Techniques des Eaux, de Gabès, Tunisia*

*^5^*ISMAR-CNR (Istituto di Scienze Marine), Via Gobetti 101, 40129 Bologna, Italy

*^6^Institut National du Patrimoine, Tunis, Tunisia*

*^7^Faculté des sciences de Sfax, université de Sfax,Tunisia*

November 2023

**Description of stratigraphy, geochemical content, Roman pottery and dating**

**Geological background of the study site**

**
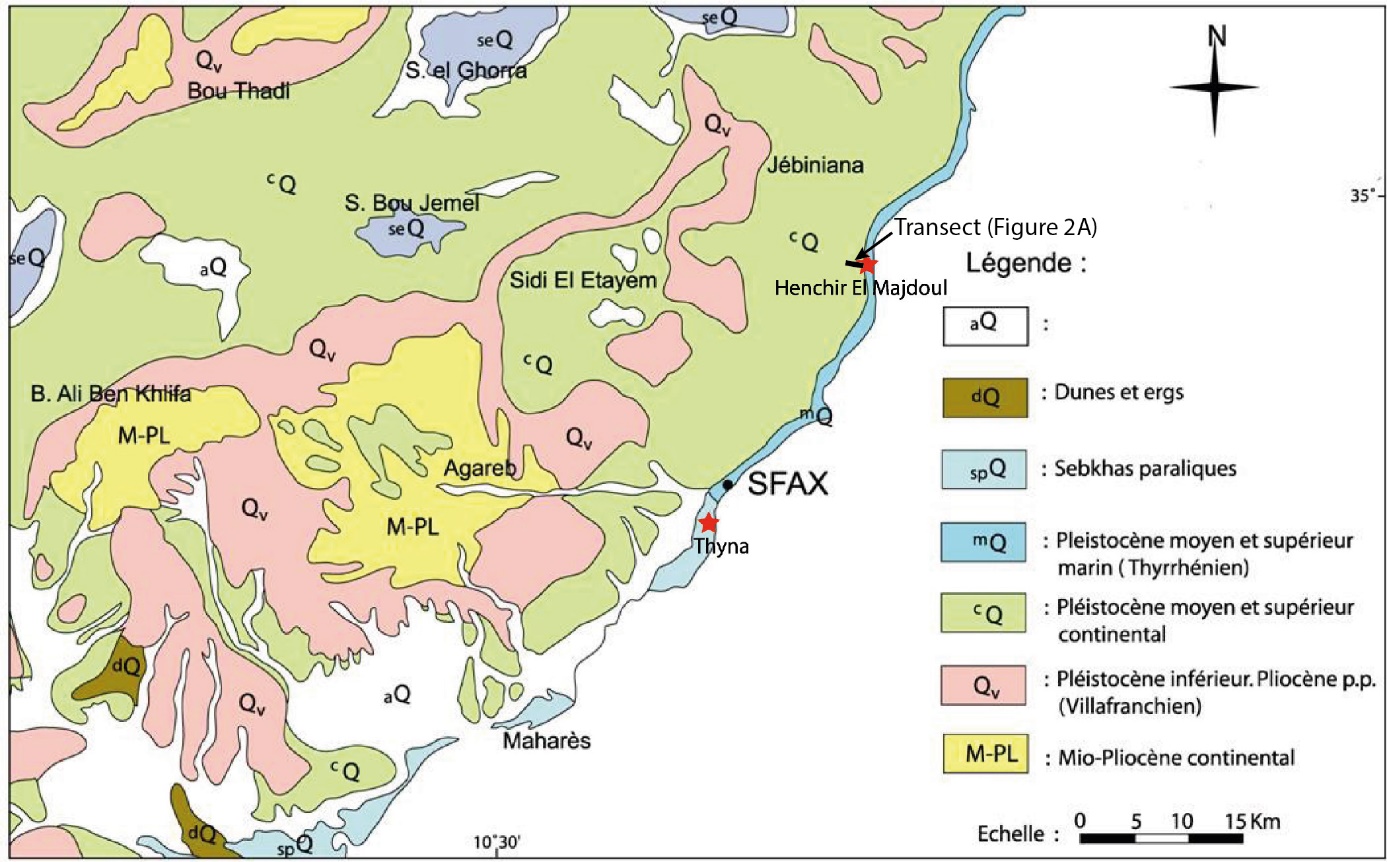
**

*Figure SM1: Geological map of the study site and chaotic deposits of Figure 2 at Henchir El Majdoul (Samir et al., 2002; Houla et al., in press; Bahrouni et al., in press). Note that the chaotic - paleotsunami outcrop covers the marine and continental late Pleistocene to Holocene deposits (^c^Q and ^m^Q) as observed on the transect of Figure 2B, and Figure SM 2. This figure is first prepared using .ai format (Adobe Illustrator for CNRS - Delegation Alsace) 2023 Adobe Systems Incorporated and its licensors* [*http://www.adobe.com/go/thirdparty_fr*](http://www.adobe.com/go/thirdparty_fr)*. This figure is also prepared using the Generic Mapping Tools (GMT version 5) cited in reference 63.*

**Lithostratigraphic units of Henchir El Majdoul :**


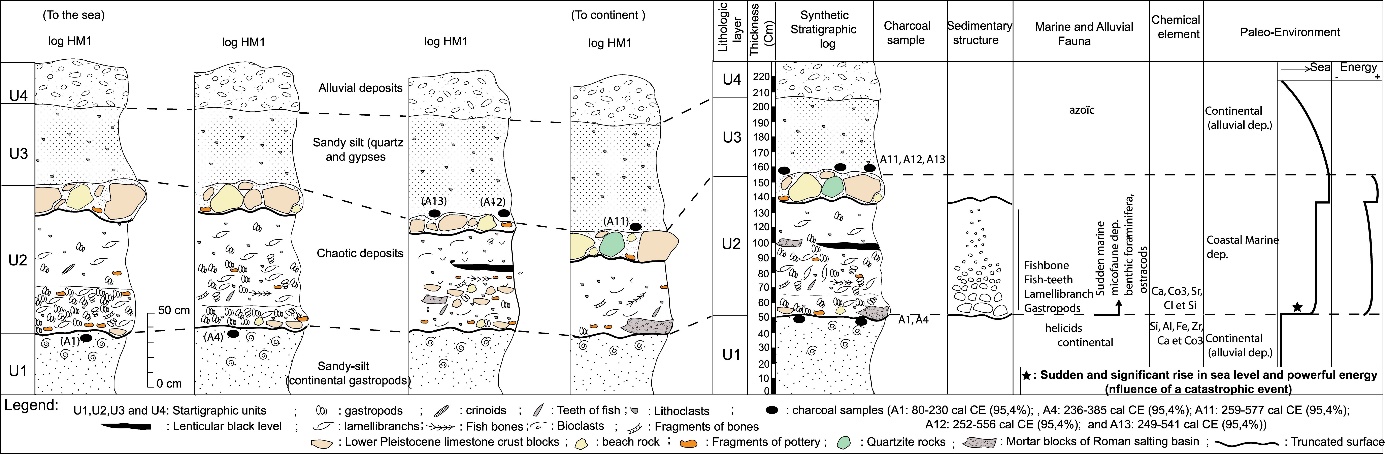


*Figure SM 2: Synthetic log of sedimentary deposits.*

We distinguish from base to top:

-Unit U1, is constituted by silts, sands, quartz dredges and rare lithoclasts and fossils of continental gastropods represented mainly by the genus Helicidae. The siliceous grains are sub-rounded to sub-angular; they are largely shiny and secondarily matte. This poor grading, these morphoscopic characteristics and the presence of a continental fauna indicate a continental alluvial environment.

-Unit U2, 40 to 70 cm thick, is composed of silty sands with quartz grains and bioclasts. This facies is rich in molluscs and lithoclasts. The stratigraphy shows that it is a chaotic deposit (has been detailed in the article).

-Unit U3, measuring 50 to 70 cm thick, is formed by a terrigenous detrital deposit, largely siliceous. It consists of silty sands with quartz dredges and showing rare lithoclasts and secondary gypsum minerals. The siliceous grains, poorly graded, are subangular to subrounded and shiny. The poor grading of this detrital material and the absence of a marine fauna indicate a continental environment (alluvial deposits).

-Unit U4, at least 30 cm thick, is composed of brown-gray azoic silts, reworking pebbles and ceramic fragments. This facies probably corresponds to alluvial deposits that probably received aeolian material.

**Geochemical analyses and isotopic dating**

Analyses of major and trace elements in the deposits of Units U1 and U2 show a significant variation in concentrations of elements of marine and terrestrial origin (Figs. 2 and SM2, Table 1).

Unit U1 is characterized by high levels of chemical elements indicative of a continental influenced environment such as Silicon (Si), Iron (Fe), Aluminium (Al) and Zircon (Zr). On the other hand, the elements of marine origin (Ca, Sr, Cl and carbonates), analysed from the samples of this unit, are characterized by low contents.

| Beta Number | Sample ID | Combustion Weight (mg) | Approximate % Carbon | d13C  o/oo | Conventional Age (pMC +/- 1 sigma) | Conventional 14C Age BCE (+/- 2 sigma) | Age  cal CE  (95.4%) |
| --- | --- | --- | --- | --- | --- | --- | --- |
| Beta - 564162 | A1 | - | - | -23.9 | 79.33 +/- 0.30 | 1860 +/- 30 | 80 - 230 |
| Beta - 564163 | A4 | - | - | -24.6 | 80.52 +/- 0.30 | 1740 +/- 30 | 236 - 385 |
| Beta-568477 | A13 | 2.1 | 65 | -25.5 | 81.23 +/- 0.30 | 1670 +/- 60 | 240 - 540 |
| Beta-568476 | A12 | 2.1 | 65 | -23.2 | 81.43 +/- 0.30 | 1650 +/- 60 | 250 - 540 |
| Beta-568475 | A11 | 2.2 | 46 | -25.6 | 81.74 +/- 0.31 | 1620 +/- 60 | 250 - 570 |

***Table SM1****: Radiocarbon dating of charcoal samples as collected below (A1 and A4) and above (A11, 12 and A13) the coastal terrace represented in Figures 2A and 3. The used calibration curve is that of* [*Reimer et al. (2020).*](javascript:go_ref('reimer2020inh','Reimer',2020,'IntCal20+Northern+Hemisphere','article'))

Geochemical analyses of samples from Unit U2 show high concentrations of Calcium (Ca), Strontium (Sr), Chlorine (Cl) and carbonates (Co3). This important enrichment of these elements clearly indicates a sudden evolution towards marine conditions and a limited continental influence. It should be noted that the high concentrations of Strontium (Sr) and Calcium (Ca) suggest a biogenic origin; indeed, the deposits of Unit U2 show a sudden and important richness in mollusc tests and marine bioclasts.


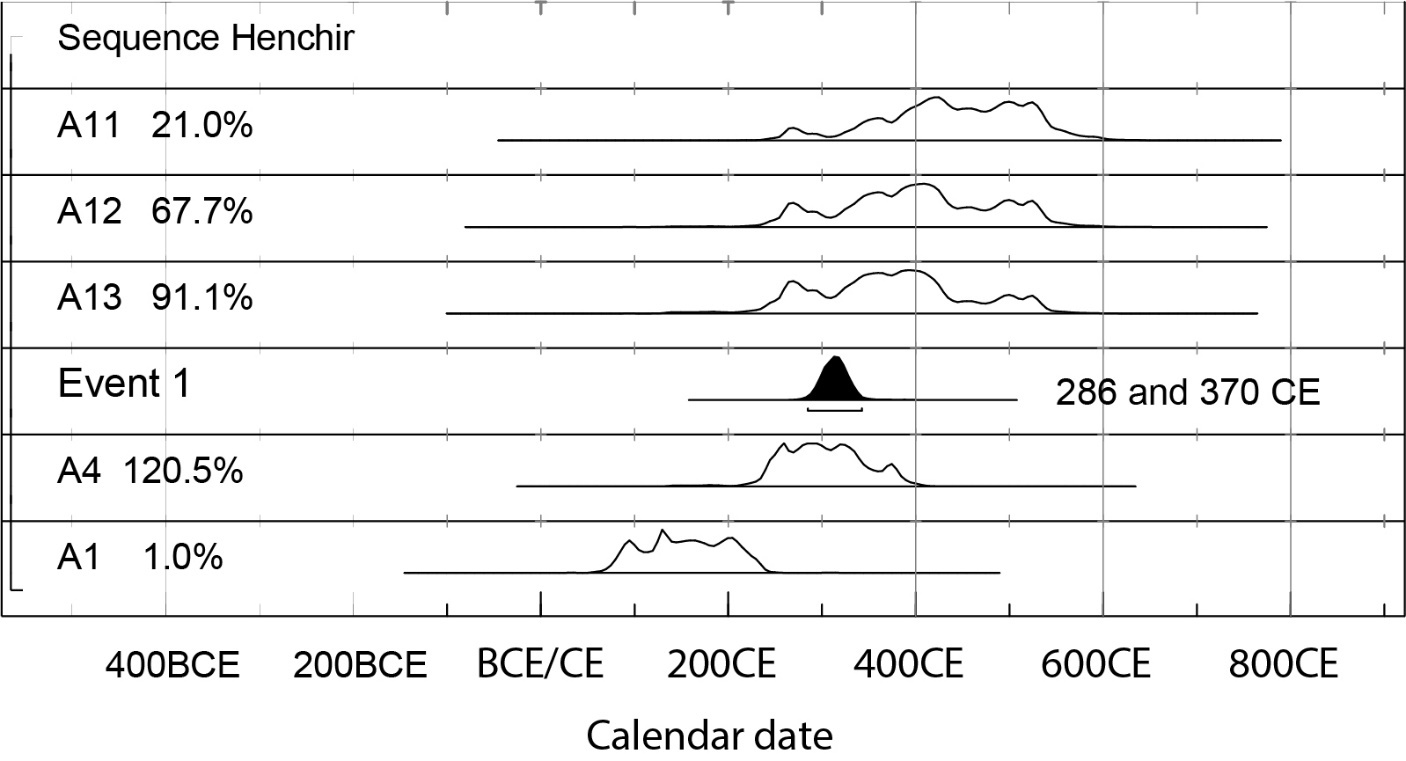


*Figure SM 3: Bayesian analysis of Oxcal calibration program (Bronk-Ramsay, 2009) with radiocarbon dating of five charcoal samples (see also Table SM1). Calibrated ages of all samples allow the bracket of Event 1 and related catastrophic deposits U2 between* 286 and 370 CE (2σ).

Several samples were collected from the stratigraphic units from which we selected 5 charcoal-rich samples in order to bracket the age of the chaotic unit U2. Samples A1 and A4 are from U1 located immediately below U2, and A11, A12 and A13 are from units U3 immediately above U2. All charcoal samples were sent for AMS dating to Beta Analytic and the obtained results in BP were calibrated using Oxcal calibration program (Bronk-Ramsey, 2009) and Intcal 20 as calibration table (Reimer et al., 2020). The Bayesian analysis in Oxcal provides an estimated dating of the chaotic deposits U2 bracketed between 236 and 541 CE (2σ age range).

**The turbidite deposits east of Malta Island**

| 1  Core and  water depth | 2  Sample name  and AMS Lab reference | 3  Position relative to the HAT | 4  Type of sample | 5  14C age  BCE  (uncalibrated) | 6  Calibrated Age (2σ) with  ΔR=147±33  (weighted mean including 2 ΔR in the region) | 7  Event age with ΔR=147±33:  age  interpolated on the top of the turbidites | 8  Likely triggering earthquakes |
| --- | --- | --- | --- | --- | --- | --- | --- |
| CALA-04  3845 m | Cala 04 VI  45.5-46.5  Poz-35787 | 23 cm above the top | Foram | 905 +/- 30 | CE 1470- 1651 | CE  1527-1814 | T2  CE 1693 Mw=7.41  (tsunamigenic) |
|  | Cala 04 V  4-5  Poz-37402 | 7 cm above the top | Foram | 1405 +/- 30 | CE 1046- 1250 | CE  1104-1417 | T3  CE 1169 Mw= 6.60  (tsunamigenic) |
|  |  |  |  |  |  |  |  |
|  | Cala 04 V  8-9  Poz-37403 | 3 cm above the top | Foram | 1860 +/- 30 | CE 598- 781 | CE 189-530 | HAT  CE 365 Crete  Mw= 8.3-8.5  (tsunamigenic) |
|  | CALA 04 IV 50  Poz-34629 | Within the coarse base | Plant | 4800 ± 35 | resedimented |  |  |

*Table SM2: Description of core CALA 1 (see location in Figure 1). Measured ages were calibrated according to the radiocarbon calibration program (Bronk Ramsey, 2009) and results are reported both for ΔR=0 (column 6) and ΔR=147±33 (column 7) calculated as the weighted mean including 2 ΔR values from published reservoir ages in the surrounding areas (Reimer et al., 2020). In the discussion we consider only the results with the ΔR=147±33 but our conclusions would not change considering ΔR=0 and correlation between HAT emplacement and the Cretan earthquake is confirmed with both ΔR values. The ages have been calculated considering the time delay introduced by sampling above the top of the megaturbidite, that is 140 ±46 years for each cm above the top (Polonia et al., 2013b).*

Three recent decimetric thick turbidites (ST1, ST2, ST3 in Fig. 4) were deposited during the last millennia in a wide area of the confined basin floor of the Ionian Sea (Polonia et al., 2013a) and they have been analysed to determine the seismic shaking and tsunami wave erosion processes that generate seismo-turbidites (Polonia et al., 2021 and references therein). Using radiocarbon and chronostratigraphic age models, these recent turbidites are correlated with major Calabrian Arc earthquakes (Fig. 4 and Table SM2, CE 1908 Messina, CE 1693 Catania, and CE 1169 Eastern Sicily respectively). These earthquakes are tsunamigenic events representing some of the strongest events ever recorded in the Calabrian Arc during historical times (Mw 7.24, Mw 7.41 and Mw 6.6 respectively, according to CPTI Working Group, 2004). The complex structure of the corresponding seismo-turbidites including a sandy coarse base and a fining upward turbidite tail (Fig. 4) is the response to the complex succession of sedimentary processes following seismic shaking and tsunami wave propagation: multiple slope failures; waning flows of the turbidity currents; water mass seiching; and tsunami backwash erosion (Polonia et al., 2017).

**Roman pottery**

At Henchir El-Majdoul, U2 contains in its 2nd unit a quantity of varied ancient pottery (Slim et al., 2004). We collected fragments of lamp, amphoras, kitchen ware (dishes and lids; Fig. SM4). There are African red slip ware, African kitchen ware, and coarse ware. Some of them are from the diagnostic:

1. A fragment of lamp type Atlante X, group C2, which preserves part of its headband, part of the disc and its elongated beak. The fragment of the disc preserves a biblical scene representing Christ trampling the serpent under his foot. The band is decorated by disks alternating with monograms. It is of the same type of Musti's lamp (Ennabli, 1976). The definition of the chronology is complex although it is common at the beginning of the 4th century (Bonifay, 2004). The type of the monogram – Chirho is attested in the form Hayes (1972), characterized by tow circles (the outer one notched) - Style A III.
2. The foot of Africana Amphora, Type III. This type is dated to the 1st third of the 4th century (Bonifay, 2004).
3. A fragment of an African kitchen ware, dish of Form (Hayes 1972), variant B (3) (see also the Marseille copy; Bonifay, 2004). This variant dates from the end of the 2nd century and the mid of the 4th century.

| **A** | **B** |
| --- | --- |
|  |  |
| **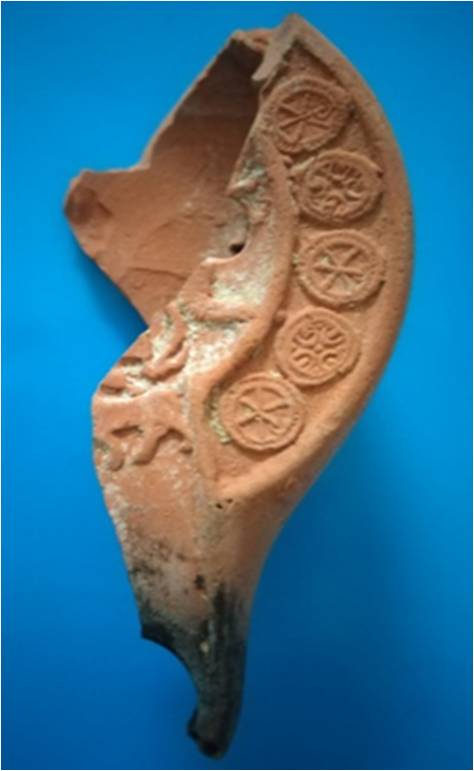** | **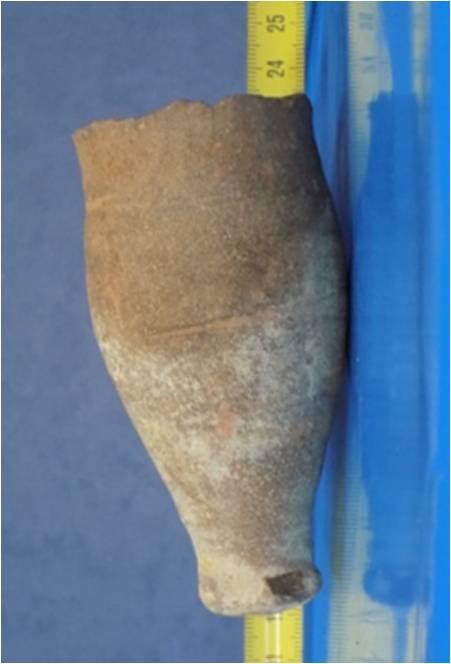** |
|  |  |

*Fig. SM 4: Example of Roman pottery fragments reworked in the chaotic deposits of U2 unit (see also stratigraphic log in Figure SM2 (Slim et al., 2004). A: Lamp Atlante X, group C Early years of 4^th^ century; B: The classic African amphora base 1st to 4th century*

We note that the archaeological material collected from the unit 2 is very homogeneous. It dates from the 4th century CE.

**Archeological and Historical evidence of damage in 4^th^ century (Table SM3)**

In Fig. SM 5, the double wall building at the archeological site of Thyna is interpreted as a paraseismic design (Slim et al., 2004). Following field investigations, we observe that only rooms intended for the dwelling can receive a painted decoration and not rooms with swimming pools (Figs SM5 and SM6). In his excavations in Thyna (Fig. 1b), Thirion (1957) pointed out an exception: numerous pieces of painted stucco from the primitive construction dated to the 3rd century, were found in the western part of Thyna city (towards the mainland), reserved for the baths. The excavations and trenches carried out by Thirion (1957) also showed that the paintings were not in situ but were piled up in the western part of the city following a significant reorganization from the east (the sea) and toward the west (the continent).

| **Site** | **location** | **Nature of damage and observations** | **References** |
| --- | --- | --- | --- |
| Neapolis  (now Nabeul) | latitude : 36.440565°  longitude : 10.721428° | - The underwater prospecting in Neapolis revealed the presence of submerged structures.  -Catastrophic event that inundated the city after a landslide in the 4th century.  -The engulfing of a part of the city is the catastrophic consequence of an extreme event that had generated, around the middle of the fourth century AD.  -The disappearance of its port and the areas dedicated to cured meats. | Fantar et al., 2019 |
| Hadrumète  (now Sousse) | latitude : 35.824647°  longitude : 10.639803° | -The wealth of the city during the high empire, but we also mention a decay of the city in the fourth century.  -The port was completely silted up and that it was disused at the end of 4^th^ | -Foucher, L. 1964  -Laporte, J. P. 2015.  -Anonymi, S. M.M. 2010. |
| Roman site of Henchir El Majdoul | latitude : 34.903255°  longitude : 10.923574° | -The stratigraphic and sedimentological sequence of the chaotic deposits dated 4th century.  - Destruction of Roman construction caused by a violent marine current | Present work |
| Thyna  (now Henchir Thyna) | latitude : 34.648616°  longitude : 10.684749° | - Severe damage and restoration that shows new houses with western migration (away from the sea) in the 4th century | - Drine, A. 2007  - Thirion, J. 1957 |
| Meninx Island  (now Djerba) | latitude : 33.684109°  longitude : 10.921065° | - Destruction of the port and inundations and damage in the second half of the 4th century.  decline of the city Meninx and the rise of the city of Girba seat of the new port in the second half of the 4th century. | -Beschaouch, A. 1986  - Slim, H. et al., 2004 |

***Table SM 3:*** *Summary table reporting the observations and nature of damaged sites dated in the 4^th^ century and all related key findings.*

| **A** | **B** |
| --- | --- |
| **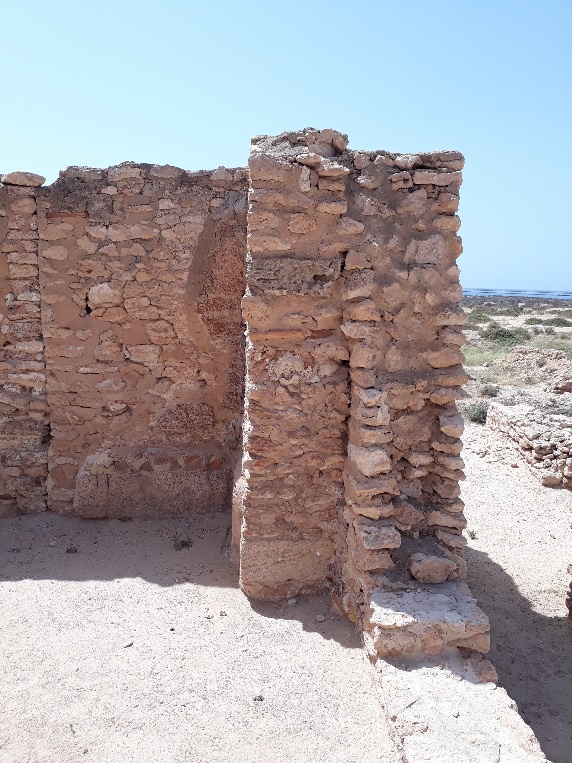** | **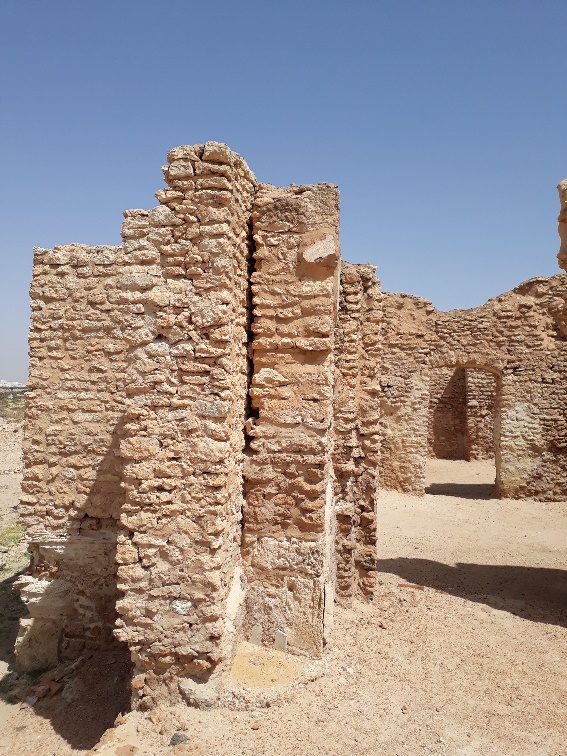** |

*Fig. SM 5:* ***A*** *– Retrofitting and retaining double-wall performed during the 4^th^ century in the archaeological city of Thyna (Thirion, 1957; Fendri, 1964).* ***B*** *– Other retrofitting and retaining double-walls located at 400 m from the coastline in the archaeological city of Thyna.*


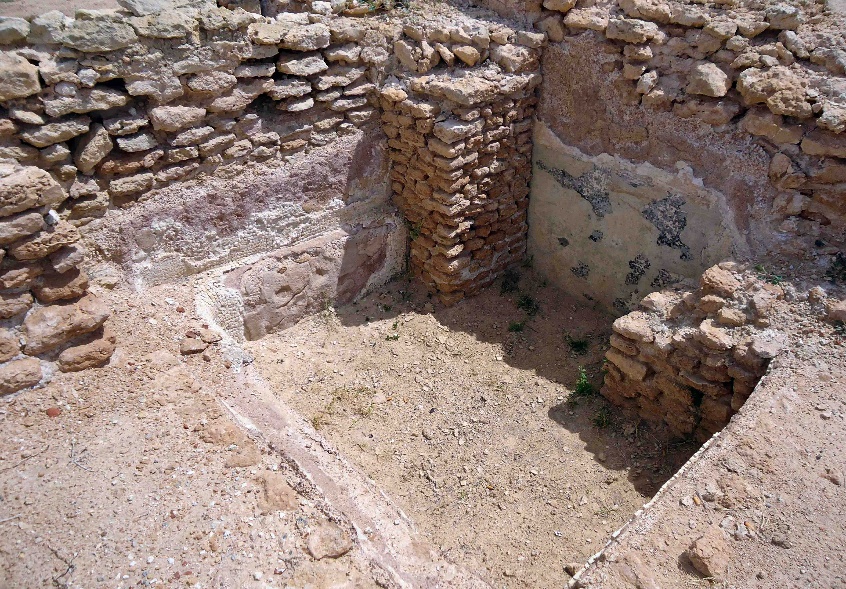


*Fig. SM 6: Thermal site in Thyna city located at 50 m from the shoreline and investigated in 1953 (Thirion, 1957). The walls and related mosaic belong to a bath and are damaged and pickled by a catastrophic event in the 4^th^ century.*

According to archaeological investigations, the city which is ~50 m from the shoreline, has been rehabilitated in the 4^th^ century (Fendri, 1985). The damage in the 4^th^ century of the city of Thyna may well be correlated with the 21 July 365 tsunamigenic earthquake.

**References Supplementary Material**

Anonymi Stadiasmus Maris Magni. (2010). In K. Müller (Ed.), Geographi Graeci minores (Cambridge Library Collection - Classics, pp. 427-514). Cambridge: Cambridge University Press. doi:10.1017/CBO9780511711176.014

Bahrouni, N., Houla, Y., Fakhraoui, M., in press. Geological Map of Tunisia, Maharès. (Editions of Geological Survey, National Office of Mines, Tunisia).

Beschaouch, A. (1986). De l'Africa latino-chrétienne à l'Ifriqiya arabo-musulmane : questions de toponymie. In: *Comptes rendus des séances de l'Académie des Inscriptions et Belles-Lettres, 130ᵉ année, N. 3,. pp. 530-549; doi :* <https://doi.org/10.3406/crai.1986.14420>

### Bonifay M. (2004). Etudes sur la céramique romaine tardive d'Afrique. British Archaeological Reports International Series 1301. Archaeopress, Oxford, 2004. ISBN 978 1 84171 651 0. <https://doi.org/10.30861/9781841716510>

[Bronk Ramsey, C. (2009). Bayesian analysis of radiocarbon dates. Radiocarbon, 51(1), 337–360.](javascript:go_ref('bronkramsey2009bar','Bronk%20Ramsey',2009,'Bayesian+analysis+radiocarbon','article'))

### CPTI Working Group (2004), Catalogo Parametrico dei Terremoti Italiani. Istituto Nazionale di Geo ﬁ sica, Gruppo Nazionale per la Difesa dai Terremoti, Storia Geo ﬁ sica Ambiente, Servizio Sismico Nazionale, Bologna.

### Drine, A. (2007). Les entrepôts de Méninx. in : Antiquités africaines, 43. L'Afrique du Nord de la protohistoire à la conquête arabe. pp. 239-251.

### Ennabli A. (1976). Lampes chrétiennes de Tunisie (musées du Bardo et de Carthage) Paris : Éditions du Centre National de la Recherche Scientifique, 334 pp., *Antiquités africaines*. [www.persee.fr/doc/etaf_0768-2352_1976_mon_1_1](https://www.persee.fr/doc/etaf_0768-2352_1976_mon_1_1).

Fantar, M., Spanu P.G., Zucc R. (2019). « Un decennio di esplorazioni nella colonia Iulia Neapolis in Africa Proconsolare », *Atti dell’Accademia Nazionale dei Lince*i, anno CDXV, *Rendiconti* se,rie IX-Volume XXIX- Fascicolo 3 - 4, Roma (2018-2019), pp. 381- 419.

Fendri, M. (1985). Cités antiques et villas romaines de la région sfaxienne. Africa 9, 151-208.

Fendri, M. (1964). Les thermes des mois à Thina. Cahiers de Tunisie, XII, 1964, p. 47-57.

Foucher, L. (1964). Hadrumetum. Paris, PUF, 1964; one vol. in-8°, 408pp. Publication of the University of Tunis, Faculty of Letters, 1st Series, vol. X.

Houla, Y., Bahrouni, N., Fakhraoui, M., in press. Geological Map of Tunisia, Sidi Salah. (Editions of Geological Survey, National Office of Mines, Tunisia).

### Hayes J. W. (1972). Late Roman Pottery, The British School at Roma, vol 1, 477 pp.

### Laporte, J. P. (2015). D'Hadrumète à Sousse, des années 350 à 859. RM2E, Revue de la mediterranée, Ed. Electronique, Tome II-1, 3-34 (in Corsican).

### Mukai T. (2016). La céramique du groupe épiscopal d'Aradi/Sidi Jdidi (Tunisie). Oxford: Archaeopress, Oxford, Series: [Roman and late antique Mediterranean pottery](https://katalog.ub.tu-braunschweig.de/vufind/Search2/Results?lookfor=%22Roman+and+late+antique+mediterranean+pottery%22&type=Series&sort=year)-9,

Polonia A, Panieri G, Gasperini L, Gasparotto G, Bellucci L.G, Torelli L., 2013a. Turbidite paleoseismology in the Calabrian Arc Subduction Complex (Ionian Sea). Geochemistry Geophysics Geosystems 01/2013; 14(1):112-140. doi:10.1029/2012GC004402

Polonia, A., Bonatti, E., Camerlenghi, A., Lucchi, R. G., Panieri, G., Gasperini, 2013b, Mediterranean megaturbidite triggered by the AD 365 Crete earthquake and tsunami, Nature Scientific Report 3, 1285 |DOI: 10.1038/srep01285.

Polonia A., Nelson H. C., Romano S., Vaiani S.C., Colizza E., Gasparotto G., Gasperini L., 2017. A depositional model of seismo-turbidites in confined basins based on Ionian Sea deposits. Marine Geology 384, 177-198.

Polonia A., C. Bonetti, J. Bonetti, M.N. Çağatay , A. Gallerani, L. Gasperini, C. H. Nelson, S. Romano, 2021. Deciphering co-seismic sedimentary processes in the Mediterranean Sea using elemental, organic carbon and isotopic data. Geochemistry, Geophysics, Geosystems, <https://doi.org/10.1029/2020GC009446>

[Reimer, P., and 41 authors (2020). The IntCal20 Northern Hemisphere radiocarbon age calibration curve (0–55 cal kBP). Radiocarbon, 62.](javascript:go_ref('reimer2020inh','Reimer',2020,'IntCal20+Northern+Hemisphere','article'))

Samir, B., Abdeljalil S., Moncef B. (2002). Geological Map of Tunisia, Agareb. (Editions of Geological Survey, National Office of Mines, Tunisia).

### Slim, H., Trousset, P., Paskoff, R., Oueslati, A., *et al.*, (2004), *Le littoral de la Tunisie, étude géoarchéologique et historique*, Études d’Antiquités africaines, CNRS éditions, Paris, 308 p.

Thirion, J. (1957). Un ensemble thermal avec mosaïques à Thina (Tunisie). In: Mélanges d'archéologie et d'histoire, tome 69, 207-245; doi: <https://doi.org/10.3406/mefr.1957.7417>.

Wessel, P., W. H. F. Smith, R. Scharroo, J. Luis, and F. Wobbe, Generic Mapping Tools: Improved Version Released, EOS Trans. AGU, 94(45), p. 409–410 (2013). [doi:10.1002/2013EO450001](http://dx.doi.org/10.1002/2013EO450001).
